# Mechanisms for Rapid Evolution of Carbapenem Resistance in a Clinical Isolate of *Pseudomonas aeruginosa*

**DOI:** 10.3389/fmicb.2020.01390

**Published:** 2020-06-19

**Authors:** Congjuan Xu, Dan Wang, Xinxin Zhang, Huimin Liu, Guangbo Zhu, Tong Wang, Zhihui Cheng, Weihui Wu, Fang Bai, Yongxin Jin

**Affiliations:** ^1^State Key Laboratory of Medicinal Chemical Biology, Key Laboratory of Molecular Microbiology and Technology of the Ministry of Education, Department of Microbiology, College of Life Sciences, Nankai University, Tianjin, China; ^2^Tianjin Union Medical Center, Nankai University Affiliated Hospital, Tianjin, China; ^3^Department of Stomatology, Tianjin First Central Hospital, Tianjin, China

**Keywords:** *P. aeruginosa*, carbapenem resistance, *oprD*, *ampC*, *ldcA*

## Abstract

Infections by *Pseudomonas aeruginosa* are difficult to cure due to its high intrinsic and acquired antibiotic resistance. Once colonized the human host, and thanks to antibiotic treatment pressure, *P. aeruginosa* usually acquires genetic mutations which provide bacteria with antibiotic resistance as well as ability to better adapt to the host environment. Deciphering the evolutionary traits may provide important insights into the development of effective combinatory antibiotic therapy to treat *P. aeruginosa* infections. In this study, we investigated the molecular mechanisms by which a clinical isolate (ISP50) yields a carbapenem-resistant derivative (IRP41). RNAseq and genomic DNA reference mapping were conducted to compare the transcriptional profiles and *in vivo* evolutionary trajectories between the two isolates. Our results demonstrated that *oprD* mutation together with *ampC* hyper-expression contributed to the increased resistance to carbapenem in the isolate IRP41. Furthermore, a *ldcA* (PA5198) gene, encoding murein tetrapeptide carboxypeptidase, has been demonstrated for the first time to negatively influence the *ampC* expression in *P. aeruginosa*.

## Introduction

*Pseudomonas aeruginosa*, as an opportunistic human pathogen, is one of the leading causes of nosocomial infections worldwide (Vincent et al., 1995). Infections by *P. aeruginosa* are difficult to treat due to its intrinsic and acquired resistance to a wide range of antibiotics, leaving limited number of effective antimicrobial agents. Carbapenems are used in clinical practice to treat *P. aeruginosa* infections. However, carbapenem resistance of clinical *P. aeruginosa* isolates has been increasingly reported (Davies et al., 2011). The mechanisms of carbapenem resistance are usually multifactorial which include: (i) acquisition of carbapenemase encoding genes through horizontal gene transfer (Poole, 2011; Potron et al., 2015), (ii) deficiency or repression of the porin (OprD) for carbapenem (Davies et al., 2011; Poole, 2011), (iii) overexpression of *mexAB-oprM* efflux pump (Poole, 2011; Liu et al., 2013; Choudhury et al., 2015), and (iv) overexpression of the chromosomal gene (*ampC*) encoding the *P. aeruginosa* intrinsic cephalosporinase (Poole, 2011; Mirsalehian et al., 2014). Although these and other studies have described the associated mechanisms of carbapenem resistance among clinical isolates of *P. aeruginosa*, there is little information on the detailed molecular mechanisms leading to the evolutionary dynamics of clinical *P. aeruginosa* isolates from carbapenems susceptibility to resistance and the impact of each of these resistance mechanisms.

In this study, we obtained two *P. aeruginosa* clinical isolates, later demonstrated to belong to the same clone, from sputum samples of the same patient with acute exacerbation of chronic bronchitis before and after treatment with biapenem. The first strain was obtained soon after the patient was admitted to the hospital while the second strain was obtained 4 days after the antibiotic treatment. The first isolate ISP50 was carbapenem susceptible, whereas the second one IRP41 was carbapenem resistant. Therefore, our goal was to decipher the molecular mechanisms by which the carbapenem resistance had been evolved so rapidly in the clinical setting. Our experimental results demonstrated that an *oprD* null mutation combined with an elevated *ampC* expression are the major contributory factors for the conversion. Furthermore, we have shown for the first time that LdcA functions as a repressor on the expression of *ampC* in *P. aeruginosa*. These findings provide novel insights into the regulatory mechanism of *ampC* expression in *P. aeruginosa*.

## Materials and Methods

### Basic Characterization of the Bacterial Strains

Bacterial strains and plasmids used in the study are listed in Table 1. Carbapenem-susceptible (ISP50) and resistant (IRP41) *P. aeruginosa* isolates characterized in this study were obtained from sputum samples of the same patient with acute exacerbation of chronic bronchitis before and after treatment with biapenem for 4 days (dosage at 0.3 g × 2/day) at the Nankai University Affiliated Hospital, Tianjin, China. The 16S rRNA encoding gene was amplified (primers listed in Supplementary Table S1) and sequenced to identify the species of these two isolates (Spilker et al., 2004). Random amplified polymorphic DNA (RAPD) typing was carried out using primer 272 as described previously (Mahenthiralingam et al., 1996). Antibiotic susceptibility test was conducted using the disk diffusion method according to the manufacturer’s recommendations, and minimum inhibitory concentration (MIC) of antibiotics was determined by the two-fold serial dilution method. Susceptibility was interpreted according to the Clinical and Laboratory Standards Institute guidelines (CLSI 2011–2018).

**TABLE 1:** Bacterial strains and plasmids used in this study.

**Strain or plasmid**	**Description**	**Source of reference**
**Strains**		
DH5α	F^–^ ϕ 80d*lac*ZΔM15 *endA1 recA1 hsdR17*(r_K_^–^ m_K_^+^) *supE44 thi-1 relA1* Δ(*lacZYA*-*argF*)*U169 gyrA96 deoR*	TransGen
S17-1	RP4-2 Tc::Mu Km::Tn*7* Tp^r^ Sm^r^ Pro Res^–^ Mod^+^	Dr. Ramphal
ISP50	Clinical isolate from sputum samples of the a female patient with acute exacerbations of chronic bronchitis before treatment with biapenem, IMP^s^	This study
IRP41	Clinical isolate from sputum samples of the a female patient with acute exacerbations of chronic bronchitis after treatment with Biapenem for 4 days, IMP^R^	This study
*Klebsiella pneumoniae* ATCC 700603	*Klebsiella pneumoniae* ATCC 700603	Fuxiang Biotechnology
PA-NK41	KPC2 carbapenemase producing *P. aeruginosa*	This study
PA14	Wild type *P. aeruginosa* strain	Liberati et al. (2006)
PA14*pbpC*::Tn	PA14 with *pbpC* disrupted by insertion of Tn	Liberati et al. (2006)
PAO1	Wild type *P. aeruginosa* strain	Kaufman et al. (2000)
PAO1Δ*ldcA*	PAO1 with *ldcA* gene deleted	This study
PA14Δ*ldcA*	PA14 with *ldcA* gene deleted	This study
ISP50Δ*ldcA*	ISP50 with *ldcA* gene deleted	This study
PAO1Δ*ampR*	PAO1 with *ampR* gene deleted	This study
**Plasmids**		
pUCP24	Shuttle vector between *E. coli* and *P. aeruginosa*; Gm^r^	West et al. (1994)
pMMB67EH-Gm	Shuttle vector between *E. coli* and *P. aeruginosa*; Gm^r^	Long et al. (2019)
pEX18Tc	Gene knockout vector, Tc^r^	Hoang et al. (1998)
pUCP24-*oprD*_ISP50_	*oprD* gene from ISP50 in pUCP24, Gm^r^	This study
pUCP24-*oprD*_IRP41_	*oprD* gene from IRP41 in pUCP24, Gm^r^	This study
pUCP24-*ampC*	*ampC* gene from ISP50 in pUCP24, Gm^r^	This study
pUC18T-miniTn7T-*ampC*-His	C-terminal His-tagged *ampC* in pUC18T-miniTn7T, Tc^r^	This study
pUCP24-*pbpC*	*pbpC* gene from ISP50 in pUCP24, Gm^r^	This study
pUCP24-*ldcA*_ISP50_	*ldcA* gene from ISP50 in pUCP24, Gm^r^	This study
pUCP24-*ldcA*_IRP41_	*ldcA* gene from IRP41 in pUCP24, Gm^r^	This study
pMMB-*ampR*_ISP50_	*ampR* gene from ISP50 in pMMB67EH-Gm, Gm^r^	This study
pMMB-*ampR*_IRP41_	*ampR* gene from IRP41 in pMMB67EH-Gm, Gm^r^	This study
pEX18-*ldcA*	*ldcA* gene deletion on pEX18Tc, Tc^r^	This study
pEX18-*ampR*	*ampR* gene deletion on pEX18Tc, Tc^r^	This study

### Plasmid Construction

For overexpression of *oprD*, a 1,802 bp *oprD*-containing fragment with its putative Shine-Dalgarno (SD) sequence was PCR amplified using ISP50 and IRP41 genomic DNA as templates (the used primers are displayed in Supplementary Table S1). The PCR products were digested with *Bam*HI and *Hin*dIII, and then ligated into a shuttle vector pUCP24, resulting in pUCP24-*oprD*_ISP50_ and pUCP24-*oprD*_IRP41_, respectively. Constructs of pUCP24-*ampC*, pMMB-*ampR*_ISP50_, pMMB-*ampR*_IRP41_, pUCP24-*ldcA*_ISP50_, pUCP24-*ldcA*_IRP41_ and pUCP24-*pbpC* were all generated using similar procedures.

For deletion of the *ldcA* gene, a 836 bp fragment immediately upstream of the *ldcA* start codon and a 913 bp fragment downstream of the *ldcA* stop codon were PCR amplified, digested with *Eco*RI-*Bam*HI and *Bam*HI-*Hin*dIII, respectively. The two fragments were then ligated into pEX18Tc that was digested with *Eco*RI and *Hin*dIII, resulting in pEX18-*ldcA*. A pEX18-*ampR* was constructed by similar manipulations. Gene knockouts in *P. aeruginosa* were carried out by conjugal transfer of these plasmids followed by selection for single crossovers and then double crossovers as previously described (Schweizer, 1992).

### Multilocus Sequence Typing

Multilocus sequence typing (MLST) was performed following a previous description (Curran et al., 2004) with minor modifications to confirm the allelic profiles of the two isolates. Briefly, genomic DNA was isolated from overnight bacterial culture with a DNA purification kit (Tiangen Biotech, Beijing, China) for use as PCR template. The internal fragments of seven housekeeping genes (*acsA*, *aroE*, *guaA*, *mutL*, *nuoD*, *ppsA* and *trpE*) were amplified by PCR and sequenced using primers described previously (Supplementary Table S1) (Curran et al., 2004). Gene sequences were then submitted to the *P. aeruginosa* MLST database^1^ for assignment of allelic numbers. A sequence type (ST) was assigned to each isolate by combining the seven allelic numbers.

### PAE-MHT Assay for Carbapenemase Test

The PAE-MHT assay was performed as described previously (Pasteran et al., 2011) with minor modifications. Briefly, an inoculum of *Klebsiella pneumoniae* ATCC 700603 was adjusted to a 0.5 McFarland turbidity standard (Mcfarland, 1907) followed by 10-fold dilution with sterile saline, and then inoculated onto the surfaces of Mueller-Hinton agar (Oxoid) plates (diameter, 10 cm) by swabbing. The plates were allowed to stand at room temperature. After 10 min, a disk of 10 μg imipenem/meropenem (Oxoid, United Kingdom) was placed in the center of each plate. Subsequently, one colony of each *P. aeruginosa* strain, grown overnight on LB agar plate, was inoculated onto the plate in a straight line from the edge of the disk to the periphery of the plate using a 1-μl loop (BOOPU Biological Technology, Changzhou, China). Presence of growth of the *K. pneumoniae* ATCC 700603 toward imipenem/meropenem disk was interpreted as carbapenemase positive.

### Total RNA Isolation, RT-qPCR and RNAseq Analysis

Bacterial overnight culture was inoculated into fresh LB medium (1:50 dilution) and grown to an optical density of 1.0 (wavelength of 600 nm). Total RNA was extracted using an RNAprep Pure Cell/Bacteria Kit (Tiangen Biotech, Beijing, China) and reverse transcribed to cDNA with random primers and PrimeScript Reverse Transcriptase (Takara). The cDNA was mixed with indicated primers (Supplementary Table S1) and SYBR premix Ex Taq II (Takara), and then quantitatively amplified in a CFX Connect Real-Time system (Bio-Rad, United States). A 30S ribosomal protein encoding gene *rpsL* was used as an internal control.

For RNAseq, total RNA was extracted as described above, quantified and qualified by Agilent 2100 Bioanalyzer (Agilent Technologies, Palo Alto, CA, United States), NanoDrop (Thermo Fisher Scientific Inc.) and 1% agarose gel. Then, the rRNA was depleted using Ribo-Zero rRNA Removal Kit (Bacteria, Illumina). The mRNA was then fragmented and reverse-transcribed. The double-strand cDNA was purified, ends repaired and ligated with adaptors. After 11 cycles of PCR amplification, the PCR products were cleaned, validated with an Agilent 2100 Bioanalyzer (Agilent Technologies, Palo Alto, CA, United States), and quantified by Qubit 2.0 Fluorometer (Invitrogen, Carlsbad, CA, United States). The resulting libraries were subjected to sequencing using an Illumina HiSeq 2500 platform with a 2 × 150 paired-end configuration.

Sequence reads were mapped onto PAO1 reference genome (NC_002516.2) via software Bowtie2 (v2.1.0). Gene expression levels were analyzed with HTSeq (v0.6.1p1). Differentially expressed genes were identified by the DESeq Bioconductor package, with the fold-change larger than 2 and *P*-value no more than 0.05 as cutoff values.

### DNA Isolation and Reference Mapping

Bacterial genomic DNA was extracted with DNA purification kit (Tiangen Biotech, Beijing, China). Fragments smaller than 500 bp were obtained from 200 ng genomic DNA by sonication (Covaris S220), followed by end treatment and adaptors ligation. Adaptor-ligated DNA fragments of about 470 bp were recovered using beads and then PCR amplified for six cycles, and the PCR products were cleaned up using beads, validated using a Qsep100 (Bioptic, Taiwan, China), and quantified by Qubit3.0 Fluorometer (Invitrogen, Carlsbad, CA, United States). Sequencing was carried out using a 2 × 150 paired-end (PE) configuration on an Illumina Hiseq instrument according to manufacturer’s instructions (Illumina, San Diego, CA, United States). The data were aligned to the PAO1 reference genome (NC_002516.2) via software BW2 (version 0.7.12). Single nucleotide variation (SNV) or InDel mutation were detected using the software Samtools (version 1.1) and the Unified Genotyper module from GATK.

### PCR and Sequencing of *oprD*, *ampR* and *ldcA*

The full-length *oprD* gene with its 105 bp upstream and 209 bp downstream region was amplified and sequenced using primers listed in Supplementary Table S1. The *oprD* gene sequence from each isolate was aligned with the *oprD* sequence of the reference strain PAO1^2^. Analysis of *ampR* and *ldcA* were conducted with similar procedures.

### Western Blot Assay

Subcultured samples from equivalent number of bacterial cells of an optical density of 1.0 (wavelength of 600 nm) were separated on a 12% SDS-PAGE. The proteins were then transferred onto a PVDF (polyvinylidene difluoride) membrane and probed with a mouse monoclonal antibody against His-tag (Cell Signaling Technology, United States) and the RNA polymerase alpha subunit RpoA (Biolegend). Signals were generated using the ECL-Plus kit (Millipore) and detected by a Biorad imager (ChemiDocXRS).

### Quantification of Biofilm

Biofilm production was measured as described previously (Li et al., 2013). Overnight culture of each bacterium was diluted 50-fold in LB broth and incubated in each well of a 96-well plate at 37°C. After 24 h, each well was washed with phosphate buffered saline (PBS, 137 mM NaCl, 10 mM Na_2_HPO_4_, 2.7 mM KCl, pH 7.4) for three times, stained with 0.1% crystal violet, and then washed three times with PBS. Then, 0.2 ml of decolorizing solution [methanol (4): acetic acid (1): water (5)] was added into each well and incubated 10 min at room temperature. Each sample was then subjected to measurement for OD*_590_* using Varioskan Flash (Thermo Scientific, Netherlands).

### Statistical Analysis

Graphpad software was used to perform the statistical analyses. The real-time qPCR results were analyzed by Student’s *t*-test (two-tailed).

## Results

### Clinical Isolates ISP50 and IRP41 Belong to the Same Clone

Sputum samples from a patient with acute exacerbation of chronic bronchitis were collected before and after treatment with biapenem for 4 days (dosage at 0.3 g × 2/day). Two mucoid isolates were selected, one before the biapenem treatment which was sensitive to imipenem (ISP50), and the other one after the biapenem treatment which showed resistance against the imipenem (IRP41). PCR amplification of the 16S rDNAs as well as their sequence analysis indicated that both of them were *P. aeruginosa* (Supplementary Figure S1A). The MLST analysis of both isolates revealed an allelic profile for *acsA*, *aroE*, *guaA*, *mutL*, *nuoD*, *ppsA* and *trpE* as 17, 5, 12, 3, 14, 4, 7, respectively, corresponding to the same sequence type, ST244. RAPD typing was further performed on the IRP41 and ISP50 isolates, which also suggested that they were of the same clone (Supplementary Figure S1B).

### Isolate IRP41 Is Resistant to Carbapenem

Imipenem susceptibilities of IRP41 and ISP50 were found different based on the preliminary analysis with VITEK automatic microbe analysis instrument (data not shown). Therefore, we further examined the imipenem susceptibilities by both MIC and disk diffusion methods, with the results shown in Table 2 and Supplementary Figure S2. Initial isolate (ISP50) was found to be susceptible to the imipenem (53.3 mm/diameter of inhibition zone), while later isolate (IRP41) was resistant to the imipenem (10.7 mm/diameter of inhibition zone), with a 32-fold increase in MIC of imipenem. Although both strains were susceptible to meropenem (another carbapenem antibiotic) according to the Clinical and Laboratory Standards Institute guidelines (CLSI 2011–2018), IRP41 displayed a smaller diameter of inhibition zone (24 mm) compared to that of ISP50 (44 mm) (Supplementary Figures S2A–D). In addition, MIC of meropenem/biapenem in IRP41 showed two-fold/eight-fold increase compared to ISP50 (as displayed in Table 2).

**TABLE 2:** MICs (μg/ml) of indicated *P. aeruginosa* strains.

**strains**	**Imipenem (μg/ml)^a^**	**Ampicillin (μg/ml)**	**Meropenem (μg/ml)^a^**	**Biapenem (μg/ml)^b^**
ISP50	0.3125	ND	3.125	0.78125
IRP41	10	ND	6.25	6.25
ISP50/pUCP24	0.3125	ND	3.125	0.78125
IRP41/pUCP24	10	ND	6.25	6.25
IRP41/pUCP24-*oprD*_ISP50_	0.625	ND	3.125	3.125
IRP41/pUCP24-*oprD*_IRP41_	10	ND	6.25	6.25
IRP41/pUCP24-*ldcA*_ISP50_	5	ND	3.125	3.125
IRP41/pUCP24-*ldcA*_IRP41_	10	ND	6.25	6.25
PAO1	1.5625	625	3.125	1.5625
PAO1Δ*ldcA*	0.78125	1250	1.5625	0.78125
PA14	1.5625	312.5	1.5625	1.5625
PA14Δ*ldcA*	0.78125	1250	0.3906	0.3906

*ND, not determined; ^*a*^Clinical Laboratory Standards Institute (CLSI) susceptibility breakpoints: imipenem, meropenem ≤ 2 μg/ml, resistance breakpoints: imipenem, meropenem ≥ 8 μg/ml. ^*b*^ no CLSI breakpoint concentrations for biapenem have been established, but previous studies suggested that MIC ≤ 4 μg/ml is sensitive and MIC ≥ 16 μg/ml is resistant (Hoban et al., 1993; Hang et al., 2018).*

Since the production of horizontally acquired carbapenem-hydrolyzing enzymes has been defined as a contributory factor in clinical *P. aeruginosa* isolates with carbapenem resistance (Potron et al., 2015), we tested if the IRP41 produced carbapenemase using a PAE-MHT assay. As the results shown in Supplementary Figures S2E,F, compared to the PA-NK41, which produces a KPC2 carbapenemase, no carbapenemase activity was observed in the IRP41 or ISP50 strain, suggesting that the carbapenem resistant phenotype of isolate IRP41 is not due to the presence of any horizontally acquired carbapenemases.

### Differential Expression of Genes Relevant to β-Lactam Resistance

**FIGURE 1:**
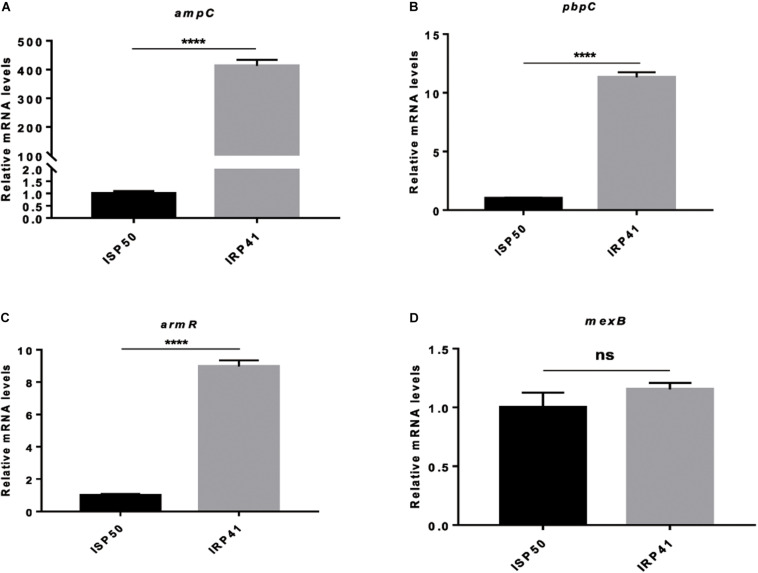
Relative mRNA levels of indicated genes in indicated strains. **(A)** Relative mRNA levels of *ampC* in indicated strains. **(B)** Relative mRNA levels of *pbpC* in indicated strains. **(C)** Relative mRNA levels of *armR* in ISP50 and IRP41. **(D)** Relative mRNA levels of *mexB* in ISP50 and IRP41. Total RNA was isolated from indicated strains at OD*_600_* of 1.0, and the relative mRNA levels of indicated genes were determined by real-time qPCR using *rpsL* as an internal control. ns, not significant, ****P < 0.0001, by Student’s *t*-test.

To elucidate the mechanism of reduced susceptibility to carbapenem in isolate IRP41, we compared the global gene expression profiles between strains ISP50 and IRP41. Expression levels of 284 genes were altered comparing the two strains (Table 3 and Supplementary Table S2). Among them, *ampC* gene, encoding the β-lactamase, displayed a 285-fold higher mRNA level in IRP41 than that in ISP50 (Table 3). To confirm the up-regulation, we further examined the mRNA levels by real-time qPCR. As shown in Figure 1A, consistent with the RNAseq result, the mRNA level of *ampC* was 413-fold higher in the strain IRP41 than that in ISP50. To further determine if the increased *ampC* expression contributed to the reduced susceptibility to carbapenem in IRP41, the *ampC* gene was overexpressed in the ISP50 strain background and the MIC of carbapenems was examined. As shown in Supplementary Table S3 and Supplementary Figure S3A, overexpression of the *ampC* (1,239-fold increase compared to that in ISP50/pUCP24) reduced the susceptibility of ISP50 to imipenem/meropenem/biapenem, with 16/2/4-fold increase in MIC. These results suggested that increased expression of the *ampC* indeed contributed to the decreased susceptibility of IRP41 to carbapenems, at least partially.

**TABLE 3:** mRNA levels of genes related to β-lactam resistance and biofilm in IRP41 compared to those in ISP50.

**Gene ID**	**Gene name**	**Gene function**	**Fold change (_IRP41/ISP50_)**	***P*-value**
PA4110	*ampC*	Beta-lactamase	285	6.42E−20
PA2233	*pslC*	Biofilm formation protein PslC	30	4.71E−09
PA2272	*pbpC*	Penicillin-binding protein 3A	8	1.49E−05
PA3719	*armR*	MexR antirepressor ArmR	5	0.001828
PA2234	*pslD*	Biofilm formation protein PslD	11	3.63E−06
PA2235	*pslE*	Biofilm formation protein PslE	16	8.15E−08
PA2236	*pslF*	Biofilm formation protein PslF	18	9.04E−08
PA2237	*pslG*	Biofilm formation protein PslG	8	4.15E−05
PA2238	*pslH*	Biofilm formation protein PslH	11	3.93E−06
PA2239	*pslI*	Biofilm formation protein PslI	10	7.32E−06
PA2240	*pslJ*	Biofilm formation protein PslJ	7	0.000124
PA2241	*pslK*	Biofilm formation protein PslK	5	0.002139
PA2242	*pslL*	Biofilm formation protein PslL	5	0.001121

Besides the *ampC*, a *pbpC* gene, encoding penicillin binding protein 3a, displayed an eight-fold up-regulation in IRP41 (Table 3). Real-time qPCR confirmed the increased expression of the *pbpC* in IRP41 (Figure 1B). However, overexpression of the *pbpC* (Supplementary Figure S3B) in ISP50 (ISP50/pUCP24-*pbpC*) had no effect on the bacterial MIC against imipenem/meropenem/biapenem (Supplementary Table S3), although it restored the MIC of carbenicillin in PA14*pbpC*::Tn to that of wild type PA14 strain (Supplementary Table S3). These results indicated that the elevated *pbpC* expression did not contribute to the carbapenem resistance in IRP41.

Our RNAseq analysis also revealed that *armR*, encoding MexR antirepressor (ArmR), was up-regulated five folds in IRP41. It had been demonstrated that MexR represses the expression of *mexAB-oprM* efflux pump (Daigle et al., 2007) whose over-expression confers meropenem resistance in *P. aeruginosa* (Okamoto et al., 2002). However, RNAseq results showed no significant difference in the transcriptional levels of the *mexAB-oprM* between the two strains, and this is confirmed by real-time qPCR (Figures 1C,D). Further sequence analysis showed that no mutation happened in the *mexR*, *armR* or *mexR*–*mexA* intergenic region of the IRP41 and ISP50 strains (data not shown).

### IRP41 Encodes an Inactive OprD

To further elucidate the molecular mechanisms of the decreased susceptibility to carbapenem in IRP41, genome reference mapping was performed to identify the accumulated mutations in the genomes of IRP41 and ISP50 in comparison with PAO1 reference genome (see text footnote 2). Between strains ISP50 and IRP41, there are 84 frameshifts (deletion/insertion) and 377 nonsynonymous single nucleotide variations (SNV) (including 17 early stops) (Supplementary Table S4). Among them, a G831A substitution in the *oprD* gene resulted in a premature termination of the OprD in the IRP41 strain. To confirm the result, *oprD* genes were PCR amplified from the genomic DNA of strains ISP50 and IRP41. Sequence analysis of the amplicons revealed that *oprD* in ISP50 was the same as that of PAO1, while the *oprD* from IRP41 showed a G831A substitution, which confirmed the reference mapping results. To assess if the G831A substitution in *oprD* contributed to the reduced susceptibility of the IRP41 to carbapenems, the *oprD* gene from both ISP50 and IRP41 were expressed in the IRP41 strain background. As can be observed in Table 2, introduction of the *oprD*_ISP50_ reduced the MIC of imipenem/meropenem/biapenem in IRP41, with 16/2/2-fold decrease, while the *oprD*_IRP41_ did not. These results indicated that the G831A substitution in *oprD* indeed disrupted its function and lead to the reduced susceptibility to carbapenem in strain IRP41.

### Mechanisms of *ampC* Hyper-Expression in IRP41

AmpR is a transcriptional regulator encoded immediately upstream of the *ampC*, regulating the *ampC* expression (Kong et al., 2005). The genome reference mapping result revealed that the *ampR* sequence of IRP41 is identical to that of PAO1, while in strain ISP50 an “A” was substituted by “G” at 821^th^ position of the *ampR* gene, resulting in an E274G substitution. These results were further confirmed by sequencing of the PCR amplicons. To test if the nonsynonymous mutation in *ampR* of ISP50 resulted in a lower level of *ampC* expression, both *ampR* genes from IRP41 and ISP50 were cloned and expressed in the background of ISP50. As shown in Supplementary Figure S3C and Supplementary Table S3, expression of the *ampR*_ISP50_ conferred ISP50 strain a higher *ampC* mRNA level and increased MIC against ampicillin compared to that of *ampR*_IRP41_, although it had no observable influence on the MIC of imipenem/meropenem/biapenem. The *ampR*_ISP50_ and *ampR*_IRP41_ were further introduced into PAO1Δ*ampR*, and their effects on MICs to imipenem, meropenem, biapenem and ampicillin in PAO1Δ*ampR* were examined. As shown in Supplementary Table S3, both *ampR*_ISP50_ and *ampR*_IRP41_ restored the MIC against ampicillin and biapenem in PAO1Δ*ampR* to that in wild type PAO1 strain, while in contrast to *ampR*_IRP41_, the *ampR*_ISP50_ conferred PAO1Δ*ampR* a repeatable two-fold increase in MIC against imipenem compared to that of wild type PAO1. Unexpectedly, no influence on MIC of meropenem was observed in PAO1Δ*ampR*. These results suggested that A821G SNV of *ampR* in fact confers slightly higher *ampC* expression-activating capacity than the wild type AmpR, thus resulting in higher, rather than lower *ampC* in ISP50.

**FIGURE 2:**
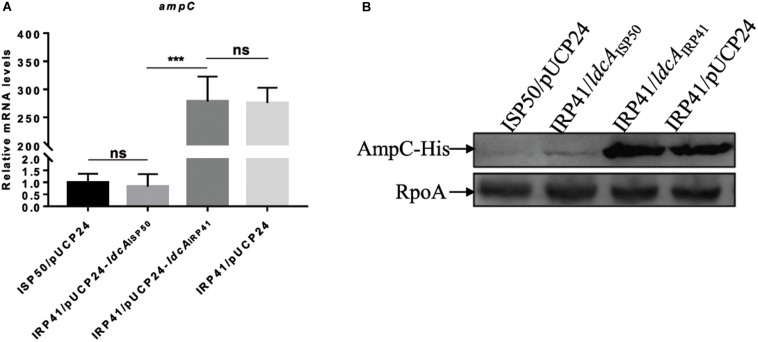
Mechanism of increased mRNA levels of *ampC* in IRP41. **(A)** Relative mRNA levels of *ampC* gene in indicated strains. Total RNA was isolated from indicated strains at OD*_600_* of 1.0, and the relative mRNA levels of *ampC* were determined by real-time qPCR using *rpsL* as an internal control. ns, not significant, ***P < 0.001, by Student’s *t*-test. **(B)** Levels of AmpC-His in indicated strains detected with Western blot assay. Strains with an *ampC*-His in their chromosomes were cultured to OD*_600_* of 1.0. Samples from equal number of bacterial cells were loaded onto 12% SDS-PAGE gels and probed with an antibody against His-tag or RpoA. The RNA polymerase alpha subunit RpoA served as a control.

*ldcA* (PA5198) encodes a cytosolic LD-carboxypeptidase which removes the terminal amino acid D-alanine from the tetra peptide of the peptidoglycan (Korza and Bochtler, 2005). Our genome reference mapping and PCR amplicons sequencing results revealed that a C was deleted at 445 bp of the *ldcA* gene in strain IRP41, leading to a frameshift mutation after R149 of the LdcA. To test if this frameshift mutation in *ldcA* contributed to the elevated expression of the *ampC* in IRP41, we expressed the *ldcA*_IRP41_ or *ldcA*_ISP50_ (same as *ldcA* of PAO1) in the IRP41 background. As shown in Figure 2A, the relative mRNA level of *ampC* in IRP41/pUCP24-*ldcA*_ISP50_, but not in IRP41/pUCP24-*ldcA*_IRP41_, was restored to that in ISP50/pUCP24, and the MIC to imipenem/meropenem/biapenem in IRP41/pUCP24-*ldcA*_ISP50_ was decreased by two folds (Table 2). To confirm the expression level of *ampC* gene, a C-terminal His-tagged *ampC* driven by its native promoter was integrated into the genomes of ISP50 and IRP41. As shown in Figure 2B, the AmpC-His level was much higher in IRP41 than that in ISP50, and overexpression of *ldcA*_ISP50_, but not *ldcA*_IRP41_, reduced the amount of AmpC-His in the IRP41 to almost that of the ISP50. These results demonstrated that frameshift-mutation of the *ldcA* in IRP41 contributes to the increased expression of the *ampC* in IRP41.

**FIGURE 3:**
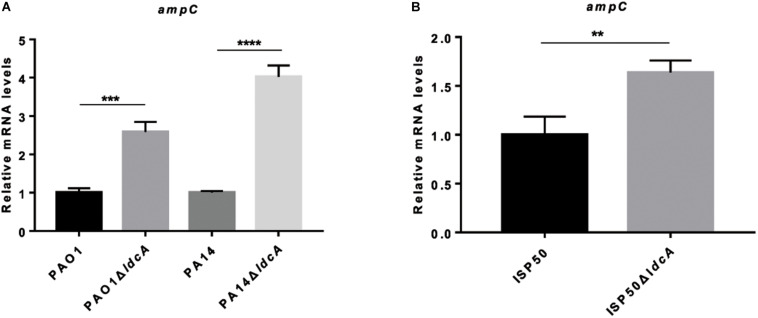
Relative mRNA levels of *ampC* gene in indicated strains **(A,B)**. Total RNA was isolated from indicated strains at OD_600_ of 1.0, and the relative mRNA levels of *ampC* gene were determined by real-time qPCR using *rpsL* as an internal control. **P < 0.01, ***P < 0.001, ****P < 0.0001, by student’s *t*-test.

To further confirm the effect of *ldcA* on *ampC* repression, the whole *ldcA* open reading frame was deleted in PA14 and PAO1 backgrounds, resulting in PA14Δ*ldcA* and PAO1Δ*ldcA*, respectively. Similar to previous report in *E. coli* (Templin et al., 1999), both of the *ldcA* deletion mutants showed a reduced growth rate and proneness to lysis during stationary growth phase (Supplementary Figure S4). Interestingly, the expression levels of *ampC* displayed 2.6-fold and 4.0-fold increase in the Δ*ldcA* mutants compared to their parental strains PAO1 and PA14, respectively (Figure 3A). Accordingly, the MIC of ampicillin in PAO1Δ*ldcA* and PA14Δ*ldcA* displayed a two-fold and four-fold increase compared to their respective parental strains (Table 2). We further deleted the *ldcA* gene from the ISP50 strain. Consistent with the above observations, the expression level of *ampC* in the ISP50Δ*ldcA* increased 1.8-fold compared to that in the ISP50 strain (Figure 3B). The difference in fold increase of *ampC* mRNA levels between ISP50 and IRP41 (413 folds) vs. between ISP50 and ISP50Δ*ldcA* (1.8 folds) suggested that other factors also contributed to the increased expression of the *ampC* in IRP41.

In addition, we also examined the effect of *ldcA* mutation on MICs against piperacillin, ceftazidime, aztreonam, ciprofloxacin and tobramycin. Except for a two-fold decrease against ceftazidime and meropenem in PAO1Δ*ldcA*, no MICs change was observed for piperacillin, aztreonam, ciprofloxacin and tobramycin in *ldcA* mutants (Table 4). However, we didn’t obtain the IRP41Δ*ldcA*, although deletion of *ldcA* in IRP41 strain had been tried for several times. We assume that it may be due to presence of some unknown factors to prevent the recombination in IRP41.

**TABLE 4:** MICs (μg/ml) of indicated *P. aeruginosa* strains.

**Strains**	**TAZ (μg/ml)**	**Cipro (μg/ml)**	**Tob (μg/ml)**	**Pip (μg/ml)**	**AZT (μg/ml)**	**Mem (μg/ml)**
PAO1/pUCP24	3.125	0.3125	8	31.25	1.953125	3.125
PAO1Δ*ldcA*/pUCP24	1.5625	0.3125	8	31.25	1.953125	1.5625
PAO1Δl*dcA*/pUCP24-*ldcA*	3.125	0.3125	8	31.25	1.953125	3.125
ISP50/pUCP24	3.125	0.625	16	62.5	7.8125	3.125
ISP50Δl*dcA*/pUCP24	3.125	0.625	16	62.5	7.8125	3.125
ISP50Δ*ldcA*/pUCP24-*ldcA*	3.125	0.625	16	62.5	7.8125	3.125

*TAZ, ceftazidime; Cipro, ciprofloxacin; Tob, tobramycin; Pip, piperacillin; AZT, aztreonam; Mem, meropenem.*

Since AmpR is the regulator of *ampC*, and its 274^th^ amino acid is different between IRP41 and ISP50, we further compared the *ampC* expression levels and the MIC to ampicillin/imipenem/meropenem/biapenem between ISP50Δ*ldcA* expressing AmpR_ISP50_ and AmpR_IRP41_. As shown in Supplementary Figure S3D, consistent with that in the ISP50 strain, expression of AmpR_ISP50_ in ISP50Δ*ldcA* (ISP50Δ*ldcA*/pMMB-*ampR*_ISP50_) resulted in a higher mRNA level of *ampC* compared to that of ISP50Δ*ldcA*/pMMB-*ampR*_IRP41_, as well as a two-fold increase in MIC of ampicillin (Supplementary Table S3). Similar to that in ISP50 strain, overexpression of both AmpR_IRP41_ and AmpR_ISP50_ showed no influence on MIC of imipenem/meropenem/biapenem in the ISP50Δ*ldcA* strain. Again, these are consistent with the earlier observation that the AmpR point mutation in ISP50 confers a higher transcriptional activator activity on *ampC*.

Furthermore, as the expression level of *pbpC* is much higher in IRP41 than that in ISP50, we overexpressed *pbpC* in the ISP50Δ*ldcA* background to see if the elevated *pbpC* expression had any effect on the *ampC* mRNA levels. However, the mRNA level of *ampC* was not affected by the overexpression of *pbpC* in ISP50Δ*ldcA* (data not shown), consistent with such null effect observed in the ISP50 background (Supplementary Table S3 and Supplementary Figure S3E).

### IRP41 Is a Better Biofilm Former Than ISP50

**FIGURE 4:**
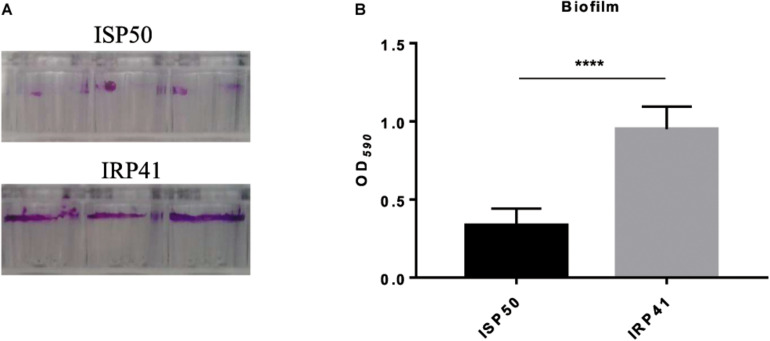
Biofilm formation by ISP50 and IRP41 strains. Overnight cultures of ISP50 and IRP41 were 50-fold diluted in LB and incubated in each well of a 96-well plate for 24 h at 37°C. Then the biofilm was stained with 0.1% crystal violet **(A)**, dissolved by 0.2 ml of decolorizing solution and subjected to measurement at OD_590_ **(B)**. ****P < 0.0001 by Student’s *t*-test.

Our RNA-seq result also revealed an increased expression of *pslD-pslL* operon (Table 3) which is involved in biofilm formation. Accordingly, we examined and compared the biofilm formation between IRP41 and ISP50 strains. As the results shown in Figure 4, the IRP41 strain produced more biofilm than the ISP50 strain.

## Discussion

In this study, we examined MICs of imipenem, meropenem and biapenem, three carbapenem antibiotics, for all the strains in Table 2 and Supplementary Table S3. Compared to meropenem, the contribution of AmpC overproduction and OprD deficiency to biapenem susceptibility in IRP41 resembles to that of imipenem. The difference among the susceptibility to imipenem, meropenem and biapenem between ISP50 and IRP41 may reflect their difference in penetration rate, stability to the hydrolysis by β-lactamase, PBP protein binding profile (Yang et al., 1995; Bonfiglio et al., 2002), β-lactamase deactivating capability and export by the efflux pumps (Chen and Livermore, 1994; Okamoto et al., 2002).

AmpC expression is regulated by AmpR, a LysR family transcriptional regulator (Kong et al., 2005). Previous studies reported that *ampR* gene point mutations were associated with increased *ampC* expression in both *Enterobacter cloacae* and *P. aeruginosa* clinical isolates (Kuga et al., 2000; Bagge et al., 2002). Our study presents E274G substitution in AmpR actually lead to a higher level of *ampC* expression. Besides the expression of *ampC*, AmpR regulates expression of hundreds of genes involved in diverse pathways such as physiological processes and metabolism (Balasubramanian et al., 2015). It has been demonstrated that *ampR* mutant displays an impaired growth under iron-limiting conditions and sensitive to many agents that affect cell growth (Balasubramanian et al., 2014, 2015). Thus, it is possible that restoration to wild type AmpR from E274G substituted AmpR of ISP50 provided the IRP41 strain a better adaption to the host environment. Also, an increased expression of the *pbpC* is neither responsible for the higher *ampC* expression nor the decreased susceptibility to carbapenem in IRP41 strain. The regulatory mechanism for the elevated *pbpC* expression in IRP41 and its functional roles remain to be investigated.

AmpD, a cytosolic peptidoglycan amidase, cleaves the peptide chain from *N*-acetyl-anhydromuramic acid peptides which are the inducer molecules for the *ampC* expression via binding to the AmpR (Langaee et al., 1998; Lister et al., 2009; Dhar et al., 2018). Mutations in AmpD and its two homologous proteins, AmpDh2 and AmpDh3, contribute to a stepwise de-repression of the *ampC* in the wild-type strain PAO1 (Juan et al., 2006). Similarly, *ampD* mutations leading to *ampC* de-repression have been reported in clinical isolates (Schmidtke and Hanson, 2008). However, some AmpC over-expressing *P. aeruginosa* strains do not exhibit mutation in *ampD*, *ampDh2*, *ampDh3*, *ampR*, or the *ampR*–*ampC* intergenic region (Wolter et al., 2007; Schmidtke and Hanson, 2008), suggesting that other undiscovered factors or pathways likely contribute to the upregulation of *ampC* expression. Besides, mutational inactivation of *dacB*, encoding penicillin-binding protein 4 (PBP4), has been reported to trigger a stable AmpC overproduction in *P. aeruginosa* (Moya et al., 2009). Mutation of the lytic transglycosylases encoding genes *sltB1* and *mltB1* in PAO1 resulted in a stable AmpC hyperproduction in the presence of β-lactam antibiotic (Cavallari et al., 2013; Juan et al., 2017). Inactivation of *mpl* (encoding cytosolic UDP-*N*-acetylmuramate:L-alanyl-γ-D-glutamyl-meso-diaminopimelate ligase) and *nuoN* (encoding NADH dehydrogenase I chain N) led to an increase in the expression of *ampC* (Tsutsumi et al., 2013; Juan et al., 2017). In the two isolates used in this study, no mutation was found in the above genes, except for the AmpR with E274G substitution in ISP50 which had no effect on the increased *ampC* expression in IRP41. These suggested the presence of unknown molecular basis driving AmpC hyperproduction in the IRP41 strain.

In the present study, inactivation of the *ldcA* contributes to the elevated expression of the *ampC* in *P. aeruginosa*. LdcA, encoding an LD carboxypeptidase, cleaves the D-alanine from *N*-acetyl-anhydromuramic acid tetrapeptides (anhNAM-P4) in *P. aeruginosa* (Korza and Bochtler, 2005), generating *N*-acetyl-anhydromuramic acid tripeptide (anhNAM-P3). Functional loss of the LdcA leads to the accumulation of anhNAM-P4, thus our observation suggests that the anhNAM-P3 is less potent inducer for the *ampC* expression than the anhNAM-P4. This is consistent with a previous study in which the anhNAM-P4 was shown to be a critical activator ligand for β-lactamase expression in *Stenotrophomonas maltophilia* (Huang et al., 2017). Results from previous studies implied anhNAM-P5 as the genuine AmpR-binding signal (Dietz et al., 1997; Lee et al., 2016) and further suggested that only muropeptides containing a terminal d-Ala-d-Ala motif (i.e., muropentapeptides) is capable of binding to the AmpR for *ampC* induction (Vadlamani et al., 2015; Dik et al., 2017). However, in a most recent report, anhNAM-P3 was demonstrated to function as an *ampC* inducer, albeit much less potent than the anhNAM-P5 (Torrens et al., 2019). As these studies were carried out under different conditions (with or without antibiotic induction) using different mutant backgrounds, it is possible that different signaling pathways may have been involved in the AmpR mediated regulation of the *ampC* in the clinical strains analyzed in this study.

This is the first report linking *ldcA* to the regulation of *ampC* expression in *P. aeruginosa*. Interestingly, compared to the 400-fold increase of *ampC* mRNA in IRP41, the Δ*ldcA* mutants of PA14, PAO1 and ISP50 showed only 1.8–4 folds increases in the *ampC* mRNA compared to their parental strains. Such minor changes in the *ampC* expression, in addition to the growth defect of the *ldcA* mutant at stationary phase, may explain why LdcA was not identified as a repressor for the *ampC* in previous studies. One may argue that the observed 2–4 folds increase in MIC to ampicillin may be due to the reduced growth of the Δ*ldcA* mutant. However, we do not feel this is the case because the proneness to lysis happens during the stationary growth phase (OD*_600_* about 2.0). In the case of IRP41, we postulate that there are other factors contributing to the increased *ampC* expression in addition to the *ldcA*. The mechanism underlying this observation is yet to be elucidated.

OprD has been reported to be the most prevalent cause of imipenem resistance among clinical isolates of *P. aeruginosa*. Insertion of various IS elements and point mutations with premature stops on the *oprD* gene had been associated with the carbapenem resistance among clinical isolates of *P. aeruginosa* (Hirabayashi et al., 2017). In this study, a G831A substitution in *oprD* resulted in loss of the OprD function. With the OprD inactivation and elevated *ampC* expression, the clinical isolate IRP41 displayed resistance to carbapenem. It has been demonstrated that derepression of AmpC in PAO1 has no obvious impact on the MIC of imipenem (Juan et al., 2006). While in another study, the loss of AmpC from PAO1 displayed a four-fold increase in susceptibility to imipenem (Masuda et al., 1999). Derepression of AmpC was associated with 4–8/8–64-fold increase in MIC of imipenem in an OprD^+^/OprD^–^ background, respectively (Livermore, 1992; Mushtaq et al., 2004). And also, mutational variants of the AmpC cephalosporinase provide *P. aeruginosa* with imipenem resistance (Rodriguez-Martinez et al., 2009). In our study, overexpression of *ampC* conferred ISP50 a 16-fold increase in MIC of imipenem. However, both AmpC and OprD are of PAO1-type in ISP50, which suggests that other unknown factors may contribute to the change in susceptibility to imipenem by the overexpression of AmpC.

MexAB-OprM is able to expel a wide variety of antibiotics (Kohler et al., 1999; Lister et al., 2009) and its expression is repressed by MexR (Poole et al., 1996). A 53-amino-acid long antirepressor, ArmR, could interact with and inhibit the MexR function, upregulating the *mexAB-oprM* expression (Wilke et al., 2008). However, in the present study, increased expression of *armR* in IRP41 did not cause altered expression level of *mexAB-oprM*. In fact, *mexAB-oprM* is expressed and contributes to antimicrobial resistance in wild type *Pseudomonas aeruginosa*, it is more difficult to detect *mexAB-oprM* overexpression in mutant cells than the other efflux pump encoding genes (e.g., *mexCD-oprJ*, *mexEF-oprN*). In addition, previous study has revealed MexR is a redox regulator which senses oxidative stress inside bacterial cells to regulate *mexAB-oprM* expression (Chen et al., 2008). *nalD* encodes a second repressor of the *mexAB-oprM* (Morita et al., 2006). Mutations in *nalD* resulted in increased MexAB-OprM expression in lab and clinical isolates of *P. aeruginosa* (Sobel et al., 2005). However, no mutation occurred in the *nalD* gene of the IRP41 or ISP50 isolates. The mechanisms underlying these observations are under active investigation. It has been demonstrated that *nalC* (PA3721), encoding a TetR family repressor, negatively regulated *armR*, and S127P substitution in NalC impaired its *armR*-repressing capacity (Cao et al., 2004). Compared to ISP50, no mutations were observed in the intergenic region between *nalC* and PA3720, while a S127P substitution in NalC happened in IRP41 (Supplementary Table S4), which may be the cause to result in the increased *armR* transcriptional level in IRP41.

IRP41 is a better biofilm former than ISP50. However, the MICs against ciprofloxacin and levofloxacin in IRP41 are the same as ISP50 (data not shown), and the MICs against gentamicin and tobramycin display a two-fold decrease in IRP41 than that in ISP50 (data not shown). A previous study had shown that the Psl did not affect bacterial MIC to biapenem for planktonic cells (Murakami et al., 2017). Therefore, we assumed that the increased biofilm production did not contribute to the increased MIC to carbapenem in IRP41.

It is not necessary for clinical isolates processing a major resistance determinant against a certain antibiotic category to accumulate the other resistance mechanisms. However, the concomitant presence of multiple carbapenem resistance mechanisms has been observed in *P. aeruginosa* (Meletis et al., 2014). In our study, the overproduction of AmpC β-lactamase together with OprD deficiency leads imipenem-susceptible ISP50 to imipenem-resistant IRP41 derivative, as well as a reduced susceptibility to meropenem and biapenem.

## Data Availability Statement

The datasets generated for this study can be found in NCBI, under accession PRJNA635437.

## Ethics Statement

We have a waiver from the ethics committee, exempting this study from the requirement to have ethics approval and written informed as the clinical strains used in the study come from the routine procedures of the clinical laboratory rather than the clinical trials.

## Author Contributions

YJ and FB conceived and designed the experiments. CX, DW, XZ, HL, GZ, and TW performed the experiments. WW, FB, ZC, and YJ analyzed the data. YJ wrote the manuscript. All authors contributed to the article and approved the submitted version.

## Conflict of Interest

The authors declare that the research was conducted in the absence of any commercial or financial relationships that could be construed as a potential conflict of interest.

## Footnotes

1 http://pubmlst.org/paeruginosa/

2 www.pseudomonas.com

## Supplementary Material

FIGURE S1 PCRresults of ISP50 and IRP41 strains. **(A)** 16S rDNA gene amplification; **(B)** RAPD typing.

FIGURE S2 Carbapenem susceptibility and carbapenemase production of indicated strains. **(A,B)** Imipenem inhibition zones of indicated strains on a 6-cm disk. **(C,D)** Meropenem inhibition zones of indicated strains on a 6-cm disk. **(E,F)** PAE-MHT assay using *K. pneumoniae* ATCC 700603 as indicator. Carbapenemase production test on imipenem **(E)** and meropenem **(F)** on a 10-cm disk, 0: ISP50, 1: IRP41, 2: PA-NK41, a producer of KPC2 carbapenemase.

FIGURE S3 Relative mRNA levels of indicated genes in indicated strains. **(A,C–E)** Relative mRNA levels of *ampC* in indicated strains. **(B)** Relative mRNA levels of *pbpC* in indicated strains. Total RNA was isolated from indicated strains at OD*_600_* of 1.0, and the relative mRNA levels of *ampC* or *pbpC* gene were determined by real-time qPCR using *rpsL* as an internal control. ns, not significant, ^∗^*P* < 0.05, ^∗∗^*P* < 0.01, ^∗∗∗^*P* < 0.001, ^****^*P* < 0.0001, by Student’s *t*-test.

FIGURE S4 Growth curves of indicated strains.

TABLE S1 Primers used in this study.

TABLE S2 Transcriptome analysis: differentially expressed genes by RNAseq without those listed in Table 2.

TABLE S3 MICs (μg/ml) of indicated *P. aeruginosa* strains.

TABLE S4 SNVs identified by genome reference mapping with elimination of SNVs with synonymous mutation and the same mutation between ISP50 and IRP41.
