# Supplementary figures and images for: Mechanisms for Rapid Evolution of Carbapenem Resistance in a Clinical Isolate of Pseudomonas aeruginosa
Source: Front Microbiol. 2020 Jun 19;11:1390. doi: 10.3389/fmicb.2020.01390 (PMC7318546; doi:10.3389/fmicb.2020.01390)

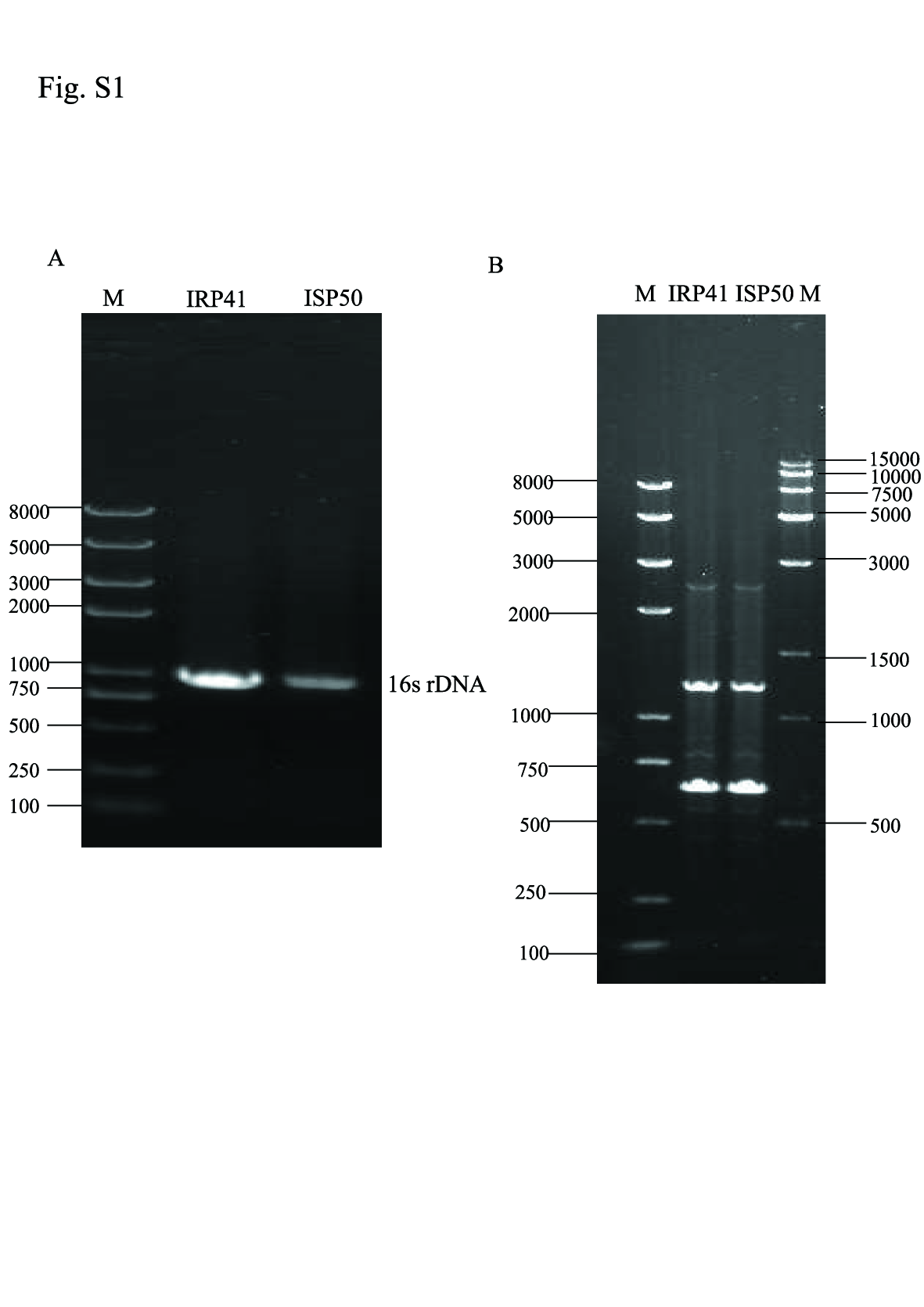

Supplement: FIGURE S1 — PCRresults of ISP50 and IRP41 strains. (A) 16S rDNA gene amplification; (B) RAPD typing. [file Image_1.TIF]

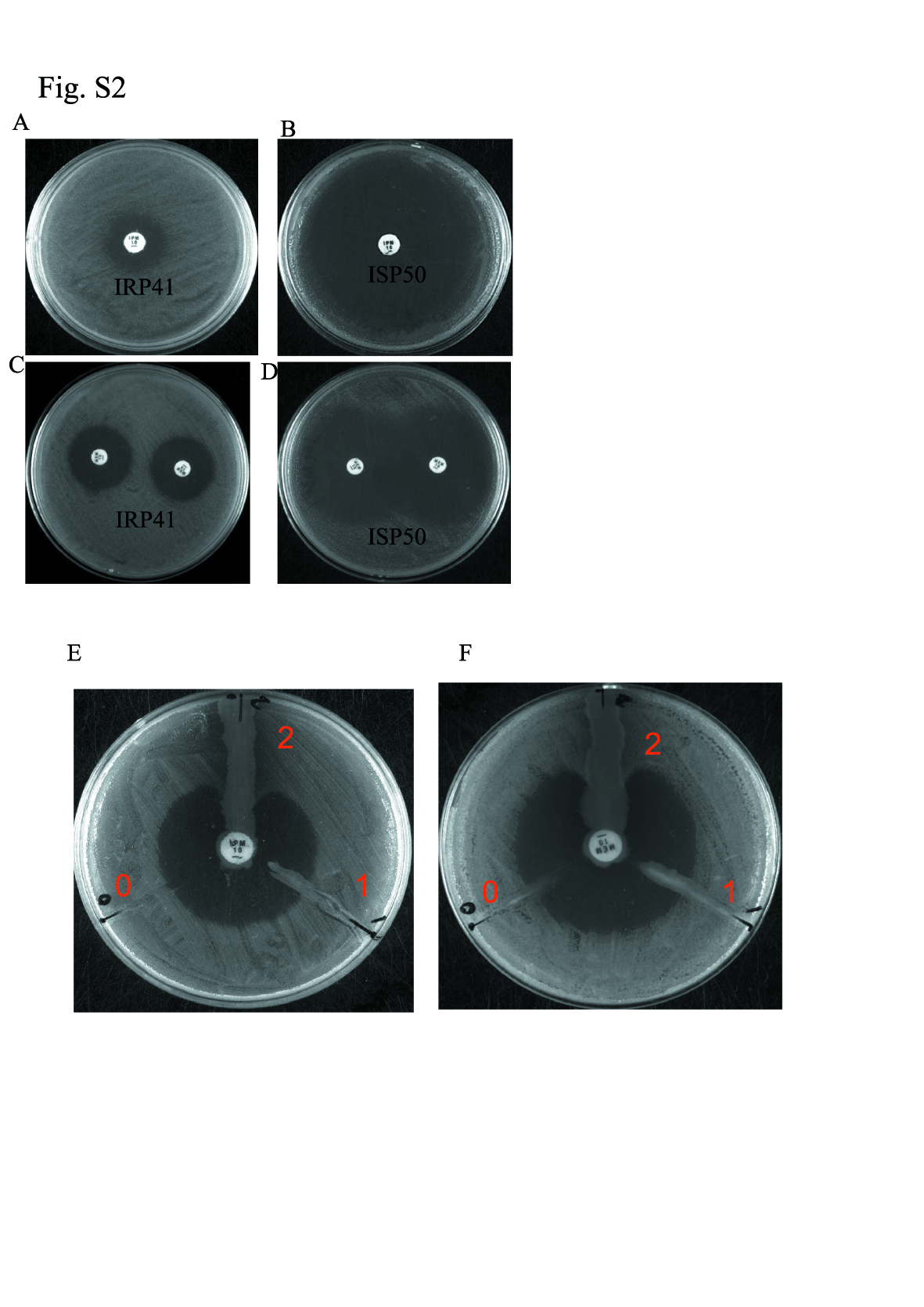

Supplement: FIGURE S2 — Carbapenem susceptibility and carbapenemase production of indicated strains. (A,B) Imipenem inhibition zones of indicated strains on a 6-cm disk. (C,D) Meropenem inhibition zones of indicated strains on a 6-cm disk. (E,F) PAE-MHT assay using K. pneumoniae ATCC 700603 as indicator. Carbapenemase production test on imipenem (E) and meropenem (F) on a 10-cm disk, 0: ISP50, 1: IRP41, 2: PA-NK41, a producer of KPC2 carbapenemase. [file Image_2.TIF]

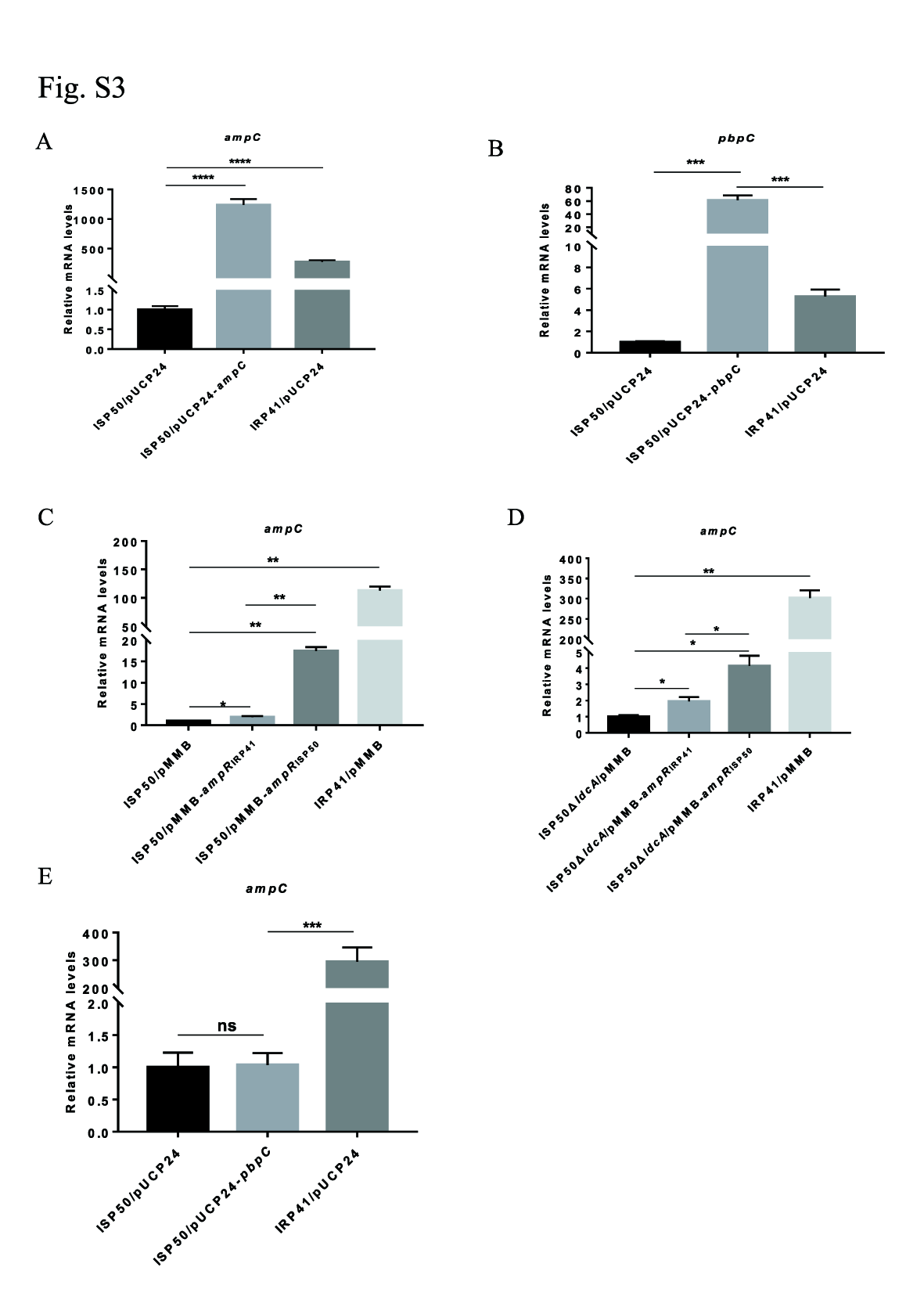

Supplement: FIGURE S3 — Relative mRNA levels of indicated genes in indicated strains. (A,C–E) Relative mRNA levels of ampC in indicated strains. (B) Relative mRNA levels of pbpC in indicated strains. Total RNA was isolated from indicated strains at OD600 of 1.0, and the relative mRNA levels of ampC or pbpC gene were determined by real-time qPCR using rpsL as an internal control. ns, not significant, ∗P < 0.05, ∗∗P < 0.01, ∗∗∗P < 0.001, ****P < 0.0001, by Student’s t-test. [file Image_3.TIF]

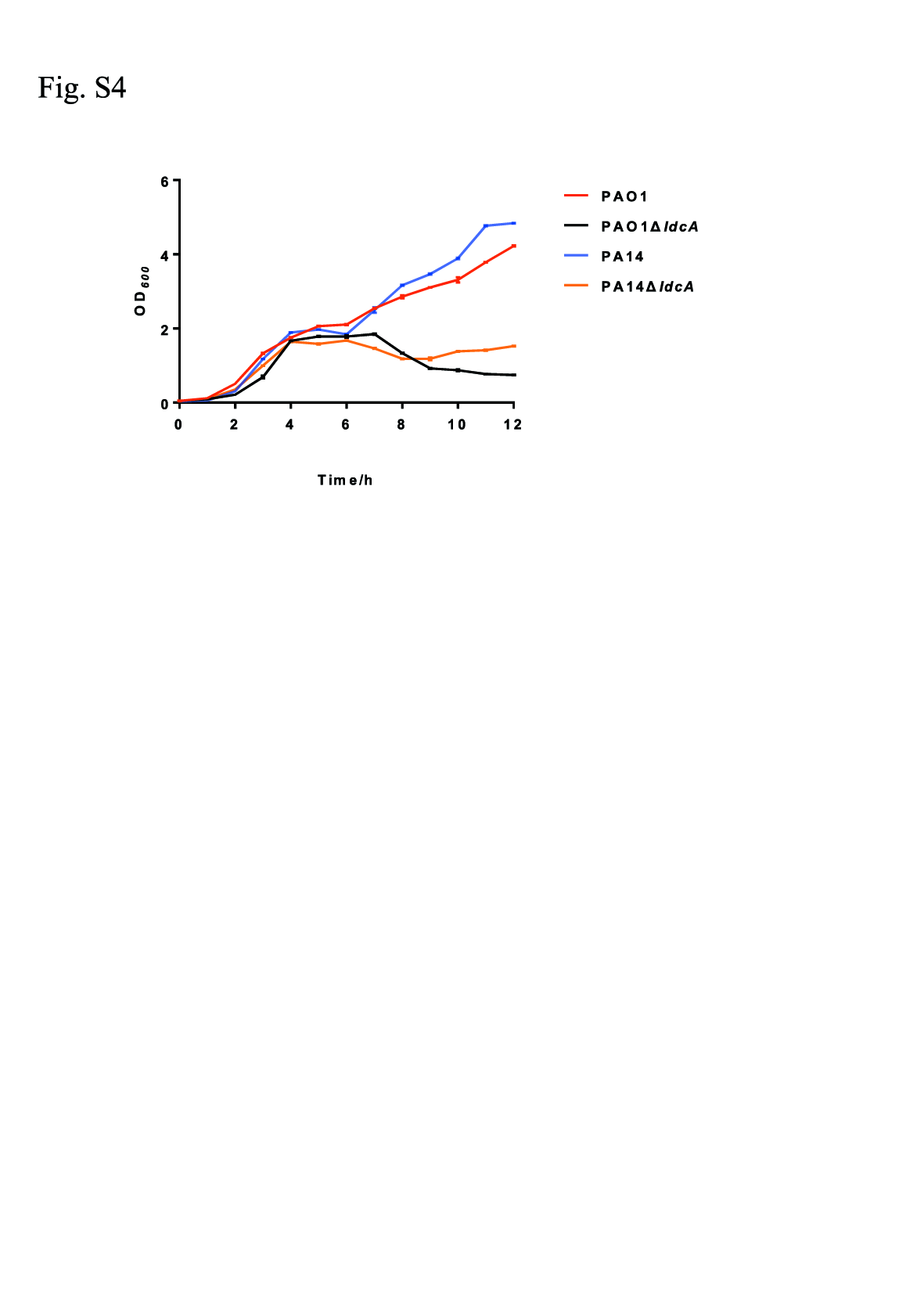

Supplement: FIGURE S4 — Growth curves of indicated strains. [file Image_4.TIF]
