# Supplementary material for: Mechanisms for Rapid Evolution of Carbapenem Resistance in a Clinical Isolate of Pseudomonas aeruginosa
Source: Front Microbiol. 2020 Jun 19;11:1390. doi: 10.3389/fmicb.2020.01390 (PMC7318546; doi:10.3389/fmicb.2020.01390)
Supplement: TABLE S1 — Primers used in this study. [file Table_1.doc]

**Table S1.** Primers used in this study.

| Primer^a^ | Sequence 5’-3’ | Use | Reference/Source |
| --- | --- | --- | --- |
| *acsA*-F | ACCTGGTGTACGCCTCGCTGAC | Gene amplification for MLST analysis | Curran *et al* (2004) |
| *acsA*-R | GACATAGATGCCCTGCCCCTTGAT |
| *aroE*-F | TGGGGCTATGACTGGAAACC |
| *aroE*-R | TAACCCGGTTTTGTGATTCCTACA |
| *guaA*-F | CGGCCTCGACGTGTGGATGA |
| *guaA*-R | GAACGCCTGGCTGGTCTTGTGGTA |
| *mutL*-F | CCAGATCGCCGCCGGTGAGGTG |
| *mutL*-R | CAGGGTGCCATAGAGGAAGTC |
| *nuoD*-F | ACCGCCACCCGTACTG |
| *nuoD*-R | TCTCGCCCATCTTGACCA |
| *ppsA*-F | GGTCGCTCGGTCAAGGTAGTGG |
| *ppsA*-R | GGGTTCTCTTCTTCCGGCTCGTAG |
| *trpE*-F | GCGGCCCAGGGTCGTGAG |
| *trpE*-R | CCCGGCGCTTGTTGATGGTT |
| *oprD*F | GCCAAGAAACACTGCGTGCTATAAG | *oprD* PCR and sequencing | This study |
| *oprD*R | AGCCCAGGCCCGAGGACCTGTTGGC |
| *dacB*F | AGACGGCCTCTTCGCGTGATGTCCG | *dacB* PCR and sequencing | This study |
| *dacB*R | CGGTCGAAGAGATCACTGGCGAAAC |
| *mltB1*F | CGCGACAGCGATCAGACAGTCCGC | *mltB1* PCR and sequencing | This study |
| *mltB1*R | GGCGAGGTTTCCGAGCAGACCGTGC |
| *mpl*F | CGCCTCCGCTGTGCTAATCTGCCG | *mpl* PCR and sequencing | This study |
| *mpl*R | GCAAGGCCACGTCGGTCTCGGTGG |
| *nuoN*F | GGTCCTGCTGGTCCTGCTCGGGGTC | *nuoN* PCR and sequencing | This study |
| *nuoN*R | GCAGGGTGCGTGGGGCGGGCCAAAG |
| *sltB1*F | GTTATGGCGGAACTTCGCTGGTTAC | *sltB1* PCR and sequencing | This study |
| *sltB1*R | AGGGGTTGGCCTTCACCGAACCGTTG |
| 16s rDNAF | GGGGGATCTTCGGACCTCA | 16s rDNA PCR/sequencing | Spilker *et al* (2004) |
| 16s rDNAR | TCCTTAGAGTGCCCACCCG |
| RAPD primer | AGGAAGGTGC | RAPD analysis  Gene sequencing for MLST analysis | Mahenthiralingam et al (1996) |
| *acsA*-SF | GCCACACCTACATCGTCTAT | Gene sequencing for MLST analysis | Curran *et al* (2004) |
| *acsA*-SR | AGGTTGCCGAGGTTGTCCAC |
| *aroE*-SF | ATGTCACCGTGCCGTTCAAG |
| *aroE*-SR | TGAAGGCAGTCGGTTCCTTG |
| *guaA*-SF | AGGTCGGTTCCTCCAAGGTC |
| *guaA*-SR | GACGTTGTGGTGCGACTTGA |
| *mutL*-SF | AGAAGACCGAGTTCGACCAT |
| *mutL*-SR | GGTGCCATAGAGGAAGTCAT |
| *nuoD*-SF | ACGGCGAGAACGAGGACTAC |
| *nuoD*-SR | TGGCGGTCGGTGAAGGTGAA |
| *ppsA*-SF | GGTGACGACGGCAAGCTGTA |
| *ppsA*-SR | GTATCGCCTTCGGCACAGGA |
| *trpE*-SF | TTCAACTTCGGCGACTTCCA |
| *trpE*-SR | GGTGTCCATGTTGCCGTTCC |
| *pbpC*OEF | GCTCTAGACTGCAACAGGATGTTTCAGCACTG | *pbpC* amplification | This study |
| *pbpC*OER | CCCAAGCTTccggcGACCAGCTTTCCGCCGAG |
| *oprD*OEF | CCCAAGCTTccggcGACCAGCTTTCCGCCGAG | *oprD* amplification | This study |
| *oprD*OER | CCCAAGCTTAGCCCAGGCCCGAGGACCTGTTGGC |
| *ampC*OEF | GCTCTAGATCCCGGGGCGGTTTCTCATGCAGCC | *ampC* amplification | This study |
| *ampC*OER | CCCAAGCTTCGCTACGCTCCGTCGCCCtcgcgag |
| *ampC*-HisF | GGGGTACCGTTCAGCGGCAAATGGGGTC | His-tagged *ampC* amplification | This study |
| *ampC*-HisR | CCCAAGCTTTCAGTGGTGGTGGTGGTGGTGGCGCTTCAGCGGCACCTTG |
| *ampR*OEF | CGGAGCTCTTTGGAGCAGAGATTGGTTCGACC | *ampR* amplification | This study |
| *ampR*OER | GCTCTAGACGTCAGCAATTCCAATCACAACCC |
| *ldcA*OEF | CGGGATCCTGAAGCATCGCCGCAACCGATGCCG | *ldcA* amplification | This study |
| *ldcA*OER | CCCAAGCTTGGCCTGATACCCGACGGGTTTGTG |
| *ldcA*UF | CGGAATTCGCTCAACGAGTCGGTCGCCATC | *ldcA* deletion | This study |
| *ldcA*UR | CGGGATCCGCGGCGATTCCGTGAAGCGTCAGG |
| *ldcA*DF | CGGGATCCGGACGGGGCGCGCCCTTCTCCGTAAG |
| *ldcA*DR | CCCAAGCTTGCCGCCTGGATCTTCGTCCGCC |
| *ampR*UF | CGGAATTCTGGTCTTCACCCCGTAGCCTTC | *ampR* deletion | This study |
| *ampR*UR | GGGGTACCCAAATGGGGTCGAACCAATCTC |
| *ampR*DF | GGGGTACCGGGGAGATAACGGTTATGCAGG |
| *ampR*DR | CGGGATCCGTCTACGACGCGATCACCTCCG |
|  |  |  |  |
| qPCR primer |  |  |  |
| q*ampC*F | GTGATGAAGGCCAATGACA | qpcr of *ampC* | This study |
| q*ampC*R | ATAGCTGAAGTAATGCGGTTC |
| q*pbpC*F | CATCAAGGTGCTGAAGAC | qpcr of *pbpC* | This study |
| q*pbpC*R | TAGGCGATGAACTGTAGT |
| q*mexB*F | GGTGAAGAACTTCCTCAT | qpcr of *mexB* | This study |
| q*mexB*R | TGTTGGAAACGATGTAGT |
| q*armR*F | GAACACTCCGCGCAACAAAC | qpcr of *armR* | This study |
| q*armR*R | CAGCTGCTCGGTGTAATCC |
| q*rpsL*F | CAAGCGCATGGTCGACAAGAG | qpcr of *rpsL* | This study |
| q*rpsL*R | ACCTTACGCAGTGCCGAGTTC |

a: F, forward; R, reverse; S, sequencing; OE, overexpression; U, upstream; D, downstream.

Curran, B., Jonas, D., Grundmann, H., Pitt, T., and Dowson, C.G. (2004). Development of a multilocus sequence typing scheme for the opportunistic pathogen *Pseudomonas aeruginosa*. *J Clin Microbiol* 42(12)**,** 5644-5649. doi: 10.1128/jcm.42.12.5644-5649.2004.

Mahenthiralingam, E., Campbell, M.E., Foster, J., Lam, J.S., and Speert, D.P. (1996). Random amplified polymorphic DNA typing of *Pseudomonas aeruginosa* isolates recovered from patients with cystic fibrosis. *J Clin Microbiol* 34(5)**,** 1129-1135.

Spilker, T., Coenye, T., Vandamme, P., and LiPuma, J.J. (2004). PCR-based assay for differentiation of *Pseudomonas aeruginosa* from other *Pseudomonas* species recovered from cystic fibrosis patients. *J Clin Microbiol* 42(5)**,** 2074-2079.
