# Supplementary material for: Mechanisms for Rapid Evolution of Carbapenem Resistance in a Clinical Isolate of Pseudomonas aeruginosa
Source: Front Microbiol. 2020 Jun 19;11:1390. doi: 10.3389/fmicb.2020.01390 (PMC7318546; doi:10.3389/fmicb.2020.01390)
Supplement: TABLE S3 — MICs (μg/ml) of indicated P. aeruginosa strains. [file Table_3.doc]

**Table S3.** MICs (μg/ml) of indicated *P. aeruginosa* strains

| strains | imipenem(μg/ml)^a^ | carbenicillin (μg/ml) | ampicillin (μg/ml) | [meropenem](javascript:;)(μg/ml) ^a^ | biapenem(μg/ml) ^b^ |
| --- | --- | --- | --- | --- | --- |
| ISP50/pUCP24 | 0.3125 | ND | ND | 3.125 | 0.78125 |
| IRP41/pUCP24 | 10 | ND | ND | 6.25 | 6.25 |
| ISP50/pUCP24-*ampC* | 5 | ND | ND | 6.25 | 3.125 |
| ISP50/pUCP24-*pbpC* | 0.3125 | ND | ND | 3.125 | 0.78125 |
| ISP50/pMMB | 0.078125 | ND | 156.25 | 3.125 | 1.5625 |
| IRP41/pMMB | 10 | ND | 5000 | 6.25 | 6.25 |
| ISP50/pMMB-*ampR*_ISP50_ | 0.078125 | ND | 312.5 | 3.125 | 1.5625 |
| ISP50/pMMB-*ampR*_IRP41_ | 0.078125 | ND | 156.25 | 3.125 | 1.5625 |
| PA14/pUCP24 | 0.78125 | 58.6 | ND | 1.5625 | 1.5625 |
| PA14*pbpC*::Tn/pUCP24 | 0.78125 | 117.2 | ND | 1.5625 | 1.5625 |
| PA14*pbpC*::Tn/pUCP24-*pbpC* | 0.78125 | 58.6 | ND | 1.5625 | 1.5625 |
| ISP50Δ*ldcA*/pMMB | 0.078125 | ND | 156.25 | 3.125 | 0.78125 |
| ISP50Δ*ldcA*/pMMB-*ampR*_IRP41_ | 0.078125 | ND | 156.25 | 3.125 | 0.78125 |
| ISP50Δ*ldcA*/pMMB-*ampR*_ISP50_ | 0.078125 | ND | 312.5 | 3.125 | 0.78125 |
| PAO1 /pMMB | 2.5 | ND | 312.5 | 3.125 | 1.5625 |
| PAO1Δ*ampR*/pMMB | 0.3125 | ND | 78.125 | 3.125 | 0.3906 |
| PAO1Δ*ampR*/pMMB-*ampR*_IRP41_ | 2.5 | ND | 312.5 | 3.125 | 1.5625 |
| PAO1Δ*ampR*/pMMB-*ampR*_ISP50_ | 5 | ND | 312.5 | 3.125 | 1.5625 |

ND: not determined; a: Clinical Laboratory Standards Institute (CLSI) susceptibility breakpoints: imipenem, meropenem ≤ 2 μg/ml, resistance breakpoints: imipenem, meropenem ≥ 8 μg/ml. ^b^: no CLSI breakpoint concentrations for biapenem have been established, but previous studies suggested that MIC ≤ 4 μg/ml is sensitive and MIC ≥ 16 μg/ml is resistant .

Hoban, D.J., Jones, R.N., Yamane, N., Frei, R., Trilla, A., and Pignatari, A.C. (1993). In vitro activity of three carbapenem antibiotics. Comparative studies with biapenem (L-627), imipenem, and meropenem against aerobic pathogens isolated worldwide. *Diagn Microbiol Infect Dis* 17(4)**,** 299-305. doi: 10.1016/0732-8893(93)90039-a.

Hang, Y., Chen, Y., Xue, L., Sun, S., Liu, L., Gao, J., et al. (2018). Evaluating biapenem dosage regimens in intensive care unit patients with Pseudomonas aeruginosa infections: a pharmacokinetic/pharmacodynamic analysis using Monte Carlo simulation. *Int J Antimicrob Agents* 51(3)**,** 484-487. doi: 10.1016/j.ijantimicag.2017.07.005.
